# P03 Identification of intervention opportunities through assessment of the appropriateness of antibiotic prescribing in surgical patients in a UK hospital using a national audit tool: a single centre retrospective audit

**DOI:** 10.1093/jacamr/dlac133.007

**Published:** 2023-01-19

**Authors:** D Hearsey, K Bamford, M Hutton, L Wade, H Coates, E Ramsay, B A Alberts, N Powell

**Affiliations:** Pharmacy Department, Royal Cornwall Hospital, Truro, TR1 3LJ; Medical Microbiology, Royal Cornwall Hospital, Truro, TR1 3LJ; General Surgery Department, Royal Cornwall Hospital, Truro, TR1 3LJ; Pharmacy Department, Royal Cornwall Hospital, Truro, TR1 3LJ; Royal Cornwall Hospital, Truro, TR1 3LJ; Royal Cornwall Hospital, Truro, TR1 3LJ; Royal Cornwall Hospital, Truro, TR1 3LJ; Pharmacy Department, Royal Cornwall Hospital, Truro, TR1 3LJ

## Abstract

**Background:**

Identifying opportunities to safely reduce antibiotic prescribing is necessary for prescribers and antibiotic stewardship teams to minimize unwarranted antibiotic use. We aimed to quantify excess antibiotic use in General Surgery.

**Methods:**

We retrospectively audited the antibiotic prescribing for patients discharged from the General Surgery specialty in an acute hospital in the south-west of England between 01/08/21 and 31/08/21 using an audit tool developed by Public Health England. The appropriateness of prescribing was determined for each patient at three antibiotic decision time-points: at initiation, the pre-72h antibiotic review, and treatment duration. Two infection specialists and a general surgeon reviewed each patient. Indication and excess days of therapy (DOTs) were calculated at each decision time-point and expressed as a proportion of total DOTs.

**Results:**

Eighty-six patients were prescribed 1162 DOTs; 192 (16.5%) excess DOTs were prescribed in 38 patients (44%), with zero excess days identified in the remaining 48 patients (56%). Seventy-five of 192 (39%) excess DOTs occurred at initiation; 55/192 (29%) after the pre-72hour antibiotic review; and 62/192 (32%) due to protracted antibiotic courses. There was concordance between the general surgeon and infection specialist for the majority of apportioned excess DOTs. However, the surgeon apportioned fewer excess DOTs 160/1162 (13.8%). Overall IV antibiotics accounted for 53.4% of total DOTs. Seventy-two of 86 (83.7%) patients received 620 intravenous DOTs; of these, 79 (12.7%) IV DOTS were unnecessary.

**Conclusions:**

We have identified excess antibiotic prescribing in General surgery with comparable excess DOTs at all three time-points.

